# Past and Present Vaccinations in Sweden

**DOI:** 10.1111/apa.70698

**Published:** 2026-09-08

**Authors:** Sven Arne Silfverdal

**Affiliations:** ^1^ Clinical Sciences, Pediatrics, Umeå University Umeå Sweden

## Abstract

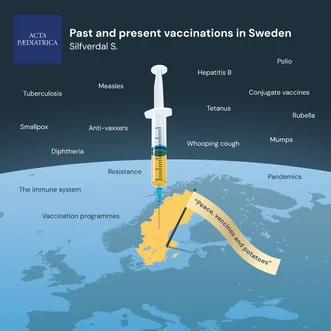

**Abbreviations**
BCG: Bacillus Calmette‐Guérin
Hib: *Haemophilus influenzae* type b
IPV: inactivated polio vaccine
MMR: measles, mumps, rubella
OPV: oral polio vaccine
WHO: World Health Organization

## 1 Introduction

Sweden's population grew rapidly during the 19th century. Esaias Tegnér (1782–1846), who was a bishop, parliamentarian and member of the Swedish Academy, had a theory about why this was happening. In 1833 he stated that this was due to ‘the peace, the vaccine and the potatoes’. That statement is still valid today. It is important that populations have good health and this requires peace, a functioning society with high compliance with vaccination programmes and good access to nutritious food. Vaccines serve little purpose if they are only available to those who can afford to pay for them or if they are received sporadically outside vaccination programmes. Therefore, well‐implemented vaccination programmes are one of the most cost‐effective ways of improving health and preventing global diseases and death. The current national vaccination programmes have been well researched, tested and effective. Diseases have largely decreased and in some cases they have completely disappeared (Figure 1).

**FIGURE 1:**
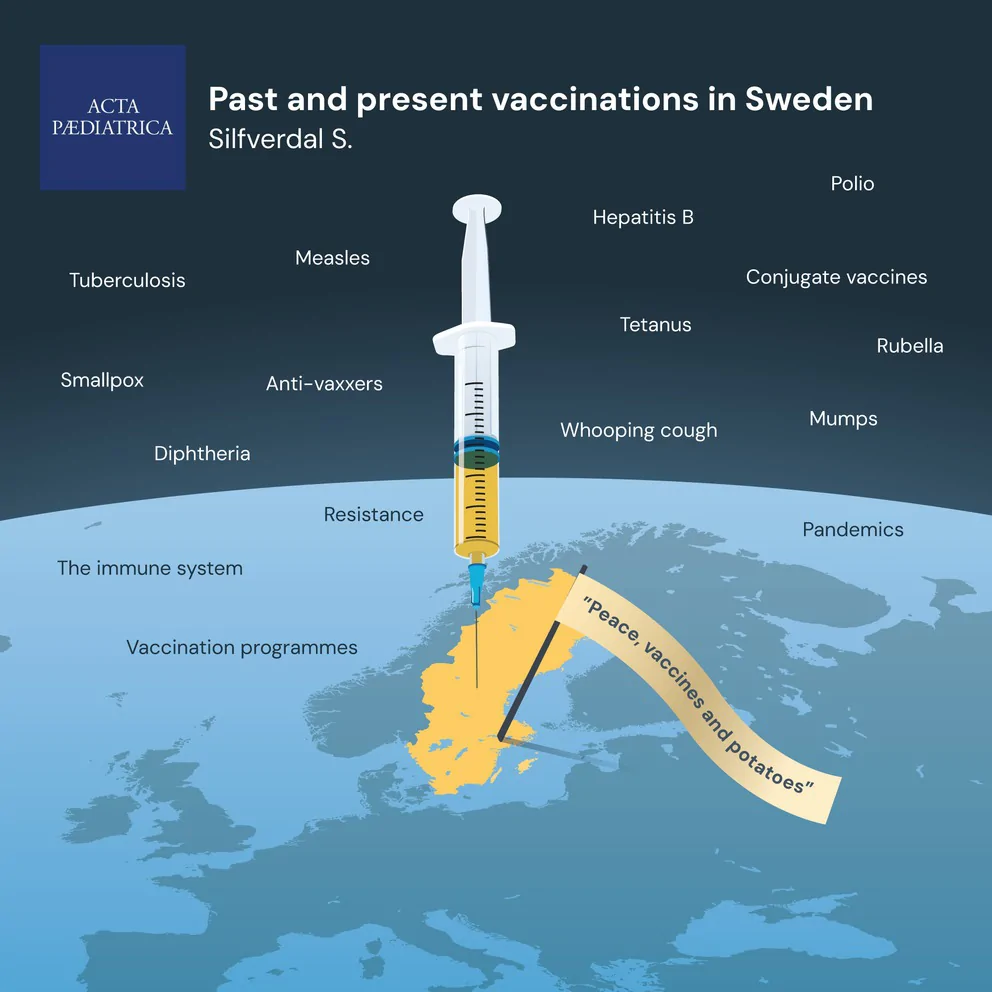
The above quote about population growth is as valid today as it was when Tegner, a member of the Swedish Academy, used it in the 19th century. Good health requires peace, high compliance with vaccination programmes and nutritious food.

During the 18th and 19th centuries, there seemed to be a greater focus on practical measures to contain the spread of diseases and epidemics, rather than ongoing research into what actually caused them in the first place. Some of the doctors, politicians and philosophers who promoted early public health approaches tried to stop epidemics by putting infected individuals into quarantine and these restrictions often lasted for at least 40 days.

They maintained that crowding, poverty and undernutrition were associated with severe outcomes. The centuries‐old miasma theory was also repeated, which maintained that epidemics were caused by bad smells and bad air, due to rotten organic material. That is why they focused on interventions to improve hygiene, such as garbage disposal, sewage systems and clean water, during these periods. Both of these approaches co‐existed in some communities [1].

However, the germ theory was presented during the second half of the 19th century, which stated that specific pathogens, such as bacteria, viruses, fungi and protozoa, caused many infectious diseases. This move away from the historical miasma theory paved the way for numerous groundbreaking medical advances, including life‐saving vaccines.

## 2 Vaccination Against Smallpox in the 19th Century

In the 5th Century BC, the ancient Greeks knew that those who survived smallpox did not get the disease again. Immunization against smallpox was already being used in China and the East Indies about 2500 years ago, after attempts were made to weaken the infectious agent in various ways. Inoculation with the smallpox virus, which was known as variolisation, was risky, unpredictable and even caused death from the virus. It was introduced in Sweden in 1754 by Professor Samuel Aurivillius (1721–1767), a prominent Swedish physician, anatomist and medical historian. However, it did not become widespread, despite the fact that Dr. Nils Rosén von Rosenstein (1706–1773) and other Swedish physicians advocated it [2].

The English doctor Edward Jenner (1749–1823) became aware that milkmaids who caught cowpox appeared immune to smallpox and he looked for an opportunity to confirm the theory. In May 1796, Jenner took pus from a cowpox lesion on a milkmaid's hand and used it to inoculate his gardener's young son. The child developed a mild case of cowpox and, when he had recovered, Jenner deliberately exposed him to human smallpox. The child showed no signs of illness and this proved that the cowpox provided full protection. In 1798, Jenner published a paper called Vaccination, after the Latin word vacca, which means cow. It showed how inoculation with cowpox could protect against smallpox [3]. This led to a general acceptance of vaccination as a better alternative to variolisation, which was very risky and dangerous.

Using the cowpox virus to vaccinate new‐born infants against smallpox started in Sweden in 1801, on a voluntary basis. Between 1801 and 1805, 13% of all new‐born infants were vaccinated and this had risen to 44% between 1811 and 1816. It became mandatory on 6 March 1816. The programme was initially supervised by the church councils and then by the parish councils from 1843 [2]. Stricter regulations were approved in 1853 and there were penalties for refusal. At the end of the 19th century, the need to modernise the legislation was discussed by the Swedish Parliament and these penalties were questioned. The theory was that statutory vaccination only defeated the purpose because it had now become generally accepted. However, many physicians disagreed, including the former provincial doctor Adolf Håkanson in Söderköping. He stated that: ‘As far as my experience goes, and I have examined records of probably more than 20 000 vaccinations, there is no reason to abandon the current mandatory vaccination. The loud outcry against this sanitary measure, which has been heard particularly from “homeopathic” quarters and has unfortunately succeeded in misleading public opinion in certain places, should, in my opinion, not lead to any other measure than to continue to maintain compulsory vaccination as a significant factor from the point of view of public health’ [2].

In 1980 the World Health Organization (WHO) declared the world free of smallpox and vaccination ceased. Until then, smallpox had been a universal disease, with a mortality rate of between 5% and 20%. The WHO's smallpox eradication initiative is still heralded as a triumph of modern medicine. This victory was partly due to international cooperation and good will and not just medical science.

## 3 How the Vaccine Against Tuberculosis Was Developed

In 1908, two French bacteriologists at the Pasteur Institute in Lille, Albert Calmette (1863–1933) and Camille Guérin (1872–1961), succeeded in cultivating a weakened strain of *Mycobacterium Bovis* . In 1921 they began testing the Bacillus Calmette‐Guérin (BCG) strain on humans for the first time.

Norrbotten County in Northern Sweden was severely affected by tuberculosis around that time. BCG vaccinations started as early as 1927 and were widespread by 1928. In 1931, Dr. Carl Naeslund published a number of papers on the BCG vaccination, which compared people who were vaccinated and unvaccinated [4]. He concluded that the BCG vaccine had a protective effect, in addition to the direct effect against tuberculosis. This so‐called non‐specific effect of vaccination is still hotly debated in vaccination circles, with regard to the value of providing live attenuated vaccines to mature the immune system. BCG vaccinations initially caused numerous side effects, but these were reduced when Arvid Wallgren around 1927 introduced a weakened vaccine that was administered intracutaneously [5]. In the 1943s, the BCG became part of the Swedish national vaccination programme and was given to all newborn infants [5]. Since 1975 it has only been given to people at increased risk.

## 4 The Emergence of Vaccines Against Diphtheria, Tetanus and Whooping Cough

The first vaccines against diphtheria, tetanus and whooping cough were introduced in the 1920s. These targeted the harmful toxins, namely poisons, of these bacteria. Alum salts started to be used as an adjuvant, which is a strengthening agent, in the vaccine. Vaccination against tetanus and diphtheria was introduced into Sweden in the 1940s. In the early 1950s, the so‐called triple vaccination, which contained vaccines against diphtheria, tetanus and pertussis in the same syringe, became available. The pertussis vaccine that was manufactured in Sweden was removed from the childhood vaccination programme in 1979, due to its poor protective effect and suspicions that it increased the risk of serious side effects. That was subsequently not confirmed. However, the vaccination only continued to target tetanus and diphtheria. During the 1980s and 1990s, large, ground‐breaking studies of a new pertussis vaccine were carried out in Sweden. At that time, it was the only country in the Western world, along with Japan, that did not have a general vaccination against pertussis. These vaccine trials led to new acellular vaccines that combined 1–5 different infectious agents and provided the same vaccine doses with good protective effect and mild side effects. As a result, the general pertussis vaccination was introduced into Sweden in 1996 and it provided good protection.

## 5 Two Different Polio Vaccines

During the 1950s, Sweden had a severe polio epidemic and many people received respiratory support treatment in iron lungs, which were the predecessors of modern respirators. In 1949, John F. Enders, Thomas H. Weller and Frederick C. Robbins discovered how to grow the poliovirus in laboratory cultures and this led to the American team being awarded the 1954 Nobel Prize in Physiology or Medicine. Jonas Edward Salk used their research to develop a killed polio vaccine (IPV, inactivated poliovaccine) in the USA in 1953 and this was licensed in the country in 1955 [6]. However, research with the killed vaccine suffered a serious setback in 1955 due to what has been called The Cutter Incident. The vaccine, which was manufactured by a California‐based family firm called Cutter Laboratories, led to 40 000 cases of polio. It left 200 children with varying degrees of paralysis and 10 died. This happened because the polio virus had not been completely killed [7]. Sven Gard also worked on a killed vaccine in Sweden that was introduced for the country‘s infants in 1957. In the USA, a live attenuated polio vaccine was developed by Albert Sabin in 1957, which could be given orally by non‐medical personnel, which lowered the cost (OPV, oral polio vaccine). It provided protection via intestinal mucosa immunity. Although the killed vaccine was widely used in the USA, it was replaced by the live attenuated vaccine that was licensed in 1961 [6]. The oral vaccine has been the used in polio vaccination campaigns by the WHO for decades.

The WHO vaccination programme now includes the IPV, which has been combined with vaccines against diphtheria, tetanus, pertussis, Haemophilus type B and hepatitis B. The number of polio cases has dramatically decreased worldwide, from more than 50 000 cases per year in 1980 to 649 cases in 2021. Although the live vaccine may have positive non‐specific healthy effects, IPV is considered safer, because OPV has been shown to be able to revert and become pathogenic. Clinical polio cases are now dominated by so‐called vaccine‐derived cases. These occur when polio vaccine strains, which have mainly originated from polio type 2, have reverted and become pathogenic, vaccine‐derived poliovirus. These then circulate and cause new polio cases. Wild polio type 2 was eradicated in 1999 and wild type 3 in 2019. However, while wild type 1 polio still exists in areas of political conflict, such as Pakistan and Afghanistan, where vaccination programmes have been unable to function. A new novel oral polio vaccine type 2 has been developed by the Global Polio Eradication Initiative to combat vaccine‐derived poliovirus 2. It is designed to be more genetically stable than the traditional monovalent OPV type 2, by reducing the risk of seeding new outbreaks but retaining its effectiveness in rapidly interrupting virus transmission [8, 9].

## 6 Vaccine Against Measles, Mumps and Rubella

During the 1960s, great progress was made in research against rubella and measles and, somewhat later, against mumps. Rubella during pregnancy can lead to severe birth defects, namely congenital rubella syndrome. Vaccination against measles, mumps and rubella was introduced into Sweden in 1982, through the combined live attenuated measles, mumps, rubella vaccine at 18 months and 12 years of age. Today it is given at 18 months and 6–8 years. As a result, domestic cases of congenital rubella syndrome have disappeared in Sweden, and the same results have been reported in Finland and the USA. Measles epidemics still occur in Europe and other global areas with poor vaccination coverage. However, the high vaccination coverage of around 97% in Sweden means that only occasional local outbreaks have occurred, but have not spread.

## 7 Combination Vaccine and Hepatitis B Vaccine

During the 1980s, new vaccines against hepatitis B and *Haemophilus influenzae* type b (Hib) were developed. Vaccination against Hib disease started in the early 1990s and this dramatically reduced meningitis, sepsis and epiglottitis due to Hib. The Hib vaccine is now included in hexavalent combination vaccines that target diphtheria, tetanus, pertussis, polio, Hib, and Hepatitis B.

## 8 Polysaccharide and Conjugate Vaccines

At 18–24 months of age, children can handle encapsulated bacteria with sugar structures on the capsule, namely polysaccharides. Vaccines made from pure polysaccharides provide limited protection and do not create any immunological memory. In addition, they do not provide herd immunity that can halt the spread of infection. This is because the immunoglobulin M antibodies that these vaccines produce are too large to pass onto the mucous membranes to prevent the bacteria from adhering. Polysaccharide conjugate vaccines work by linking the polysaccharide infectious agent to a protein, which leads to an interaction between B cells and T cells. This elicits immunoglobulin G that can pass across the mucous membranes and produce herd effects. Infants can respond well to these kinds of conjugate vaccines and obtain immunity. The first conjugate vaccine that was tested was the Hib polysaccharide conjugate vaccine, which was studied in Finland in the 1980s by Peltola and others.

The first pneumococcal vaccine was a plain polysaccharide that was tested in South Africa in 1911 and 1912 by giving it to miners. It produced relatively good efficacy, despite deficiencies in the researchers' knowledge about the infectious agents, but the vaccine was not marketed [10]. During the 1970s, a polysaccharide vaccine against 23 pneumococcal types came onto the market and in 2000, the first conjugate pneumococcal vaccine was launched. Prevenar targeted seven of the 100 plus different pneumococcal serotypes, which have varying levels of aggressiveness. More serotypes have been included in pneumococcal conjugate vaccines, but the immunogenicity was shown to decrease as the numbers of serotypes increased. Internationally, we now have vaccines containing 1‐, 15‐, and 20‐valent for infants. A polysaccharide targeting 23 serotypes has been approved for adults and for children over 2 years of age, as well as a 21‐valent conjugate vaccine. Conjugate vaccines that provide protection against up to 30 serotypes are under development.

## 9 Vaccination Programmes for Children Are Evolving Over Time

The latest vaccines to be included in childhood vaccination programmes Table 1 are against human papillomavirus, rotavirus and varicella and these have proven effective [11]. A live attenuated varicella vaccine for children is available, but studies are underway on a killed vaccine, mainly for at‐risk individuals with impaired immune function. In September 2025, Sweden introduced a protection against respiratory syncytial virus for newborn infants, with specific monoclonal antibodies providing so called passive immunisation. There is also a licensed respiratory syncytial virus vaccine that can be given to pregnant women during the third trimester, which protects their new‐born infants via maternal antibodies. However, no licensed vaccine for infants has been developed so far due to safety issues. Trials with a vaccine candidate to infants based on mRNA technology showed that those receiving the vaccine developed a severe RSV compared to those getting placebo [12].

**TABLE 1:** Current and Previous Swedish Vaccination Programmes

Vaccine against	Dates available	Programme
Smallpox	1816‐1976	Mandatory for entire population until disease was eradicated
		
Tuberculosis	1940s‐1975	1 dose for newborn infants
	1940s‐1986	1 booster dose at 14‐15 years
	1986 (ongoing)	Risk groups only
		
Diphtheria, tetanus	1940s‐1986	3 doses between 3‐12 months
	1965‐1977	1 booster dose at 7‐8 years
	1977‐2012	1 booster dose at 10 years for children born up to 2001
	1986 (ongoing)	3 doses at 3, 5 and 12 months
	2007 (ongoing)	2 booster doses at 5‐6 and 14‐16 years for children born from 2002
		
Pertussis (whooping cough)	1950s‐1979	3 doses between 3‐12 months
	1996 (ongoing)	3 doses at 3, 5 and 12 months
	2005‐2012	1 booster dose at 10 years for children born up to 2001
	2007 (ongoing)	2 booster doses at 5‐6 and 14‐16 years for children born from 2002
		
Polio	1957‐1986	3 doses between 9‐18 months
	1957‐1977	1 booster dose at 7‐8 years
	1986 (ongoing)	3 doses at 3, 5 and 12 months
	1977 (ongoing)	1 booster dose at 5 years
		
Measles	1971‐1982	1 dose after 18 months
Rubella	1974‐1982	1 dose for girls at 12 years
Measles, mumps, rubella	1982‐2007	2 doses at 18 months and 12 years for children born up to 2001
	2007 (ongoing)	2 doses at 18 months and 6‐8 years for children born from 2002
		
*Haemophilus influenzae* type B	1993 (ongoing)	3 doses at 3, 5 and 12 months
		
Hepatitis B	1996 (ongoing)	For risk groups
	2016 (ongoing)	Recommended for all infants: 3 doses at 3, 5 and 12 months
		
Pneumococcal disease	2009	3 doses at 3, 5 and 12 months
		
Human papillomavirus	2010‐2014	3 doses for girls aged 11‐13 born from 1999
	2015‐2020	2 doses for girls aged 11‐12
	2020 (ongoing)	2 doses for girls and boys aged 11‐12 (boys born from 2009)
		
Rotavirus	2019 (ongoing)	2 or 3 doses from 6 weeks
		
Varicella	Start date of January 2027	2 doses at 18 months and 6‐8 years born from 1 July 2025. Catch‐up vaccination up to 18 years.

## 10 The Role the Immune System Plays in Vaccination

The specific adaptive immune system, with antibodies and cellular immunity, targets the antigens that cause the disease. The innate immune system also plays an important role. Studies have increasingly suggested that the innate defence can be trained and stimulated to increase its effectiveness. This mechanism may help to explain the nonspecific effects of live vaccines, including reduced morbidity and death, as well as its specific effect against the targeted disease.

A number of scientific advances will make vaccine development more precise and faster than approaches to date, including new adjuvants and platforms. They also include reverse vaccinology, which is an advanced vaccine design approach based on bioinformatics and genomic sequencing. This works by identifying potential vaccine candidates directly from a pathogen's genetic code [13], together with messenger ribonucleic acid techniques. Earlier research on influenza using messenger ribonucleic acid led to successful COVID‐19 vaccines. The same approach could be used against future devastating microbial agents. It is important that vaccine trials and clinical testing continue to provide safe vaccinations.

## 11 Vaccination Coverage and Resistance

The vaccination rates in Sweden and other Nordic countries are above 97%, which is very high by international standards. This is despite, or thanks to, the great deal of trust that people have in the vaccination programme. At the turn of the last millennium, vaccination coverage for the MMR vaccine decreased due to unfounded suspicions that it could cause autism. Brian Deer, a journalist at the British Medical Journal, reviewed the research behind a Lancet paper that claimed that there was a connection between the MMR and autism and discovered that it was based on false information [14]. The paper was retracted and the lead author, Andrew Wakefield, was struck off the UK's General Medical Register. Since then, careful epidemiological studies have reinforced the fact that there is no connection between the MMR and autism. For example, they found that the siblings of children with autism did not face an increased risk of autism after MMR vaccinations.

Knowledge about the dangers of diseases has been forgotten as they have been eliminated. In the Western world, this has led to more concern and fear about the side effects of vaccination than fear of the diseases and their consequences. The vaccinations we administer today do have side effects, but they are greatly outweighed by the benefits and safety.

Hesitation and resistance are greater in some other countries than in Sweden. A survey by the Public Health Agency of Sweden in 2016 showed that 79% of parents in Sweden accepted the vaccination programme in its entirety without further questions. Another 17% expressed some concern and hesitation, but they let their children be vaccinated. Of the remaining parents, 2% were completely unresponsive to the arguments put forward by medical professionals, while 2% would consider vaccination after discussions and sometimes with modified vaccination schedules [15].

The Pandemrix vaccine and the risk of narcolepsy have been cited by some as reasons for general resistance to vaccination. Pandemrix was developed by GlaxoSmithKline to combat H1N1 influenza, which spread during 2009 and 2010. The WHO and the European Union classified H1N1 as a serious pandemic and the latter approved vaccines before normal large‐scale vaccine trials had been carried out. Pandemrix was later shown to be linked to an increased risk of narcolepsy in children and young people. This side effect could not have been captured by studies conducted before the large‐scale vaccination, because the risk was rare and millions of doses would have been required to confirm a link. The H1N1 influenza disease itself can also result in narcolepsy, as was noted in China. The viral protein in the Pandemrix vaccine had greater similarities to the wild H1N1 virus than the protein in the Arepanrix vaccine. This vaccine, which was also developed by GlaxoSmithKline, was used in North America and contained the same ASO3 adjuvant as Pandemrix [16]. This suggests that the nature of the infectious agent was a decisive factor in the onset of narcolepsy, together with the individual's genetic makeup.

## 12 Conclusion

We are facing new challenges. Vaccine development has made great progress and our understanding of the different risks and mechanisms of action has increased. At the same time, we must be vigilant and learn from events such as those described above. It is important to have vaccine registers, like the one in Denmark, so that we can effectively follow any side effects over time [17]. Such registers also enable researchers to investigate suspected connections between vaccines and unwanted side effects, in order to make vaccinations even safer.

It is worth recalling the importance of ‘peace, vaccines and potatoes’ that Esaias Tegnér mentioned in 1833, and that well‐implemented vaccination programmes are one of the most cost‐effective ways of promoting health and preventing disease and death. The vaccines we use in childhood vaccination programmes are well researched and tested. They are effective and the diseases they target have significantly decreased and, in some cases, even disappeared completely [18].

## Author Contributions

**Sven Arne Silfverdal:** conceptualization, investigation, writing – review and editing, methodology, writing – original draft.

## Funding

The author has nothing to report.

## Conflicts of Interest

The author has been involved in more than 20 vaccine trials for different companies since the mid‐1980s. He has also been a member of the advisory boards for AstraZeneca, GlaxoSmithKline, MSD, Sanofi Pasteur and Pfizer on influenza, the respiratory syncytial virus and hexavalent, meningococcal and pneumococcal vaccines.

## Data Availability

The data that support the findings of this study are available from the corresponding author upon reasonable request.
